# Paclitaxel and etoposide co-loaded polymeric nanoparticles for the effective combination therapy against human osteosarcoma

**DOI:** 10.1186/s12951-015-0086-4

**Published:** 2015-03-21

**Authors:** Bing Wang, Xiu-Chun Yu, Song-Feng Xu, Ming Xu

**Affiliations:** Department of Orthopeadic, The General Hospital of Jinan Military Commanding Region, No. 25 Shifan Road, Tianqiao District, Jinan, Shandong 250031 China

**Keywords:** Osteosarcoma, PLGA NP, apoptosis, paclitaxel, etoposide

## Abstract

**Background:**

The combination of chemotherapeutic drugs with different pharmacological action has emerged as a promising therapeutic strategy in the treatment of cancers. Present study examines the antitumor potential of paclitaxel (PTX) and etoposide (ETP)-loaded PLGA nanoparticles for the treatment of osteosarcoma.

**Results:**

The resulting drug-loaded PLGA NP exhibited a nanosize dimension with uniform spherical morphology. The NP exhibited a sustained release profile for both PTX and ETP throughout the study period without any sign of initial burst release. The combinational drug-loaded PLGA NP enhanced the cytotoxic effect in MG63 and Saos-2 osteosarcoma cell lines, in comparison to either native drug alone or in cocktail combinations. Additionally, NPs showed an appreciable uptake in MG63 cells in a time-based manner. Co-delivery of anticancer drugs resulted in enhanced cell cycle arrest and cell apoptosis. The results clearly showed that combinational drugs remarkably improved the therapeutic index of chemotherapeutic drugs. The greater inhibitory effect of nanoparticle combination would be of great advantage during systemic cancer therapy.

**Conclusion:**

Taken together, our study demonstrated that PTX-ETP/PLGA NP based combination therapy holds significant potential towards the treatment of osteosarcoma.

## Background

Osteosarcoma (OS) is one of the most prevalent malignant bone cancers affecting adolescents aged between 10 and 24 years [1]. Children and adolescent constitutes 60% of overall bone tumor cases in past 2 decades [2]. Although significant progress have been made in the treatment of OS (5 year survival rate to 65%), metastatic cancer which constitutes to only 20% survival rate is a big cause of concern [3]. The standard treatment protocol for this disease mainly includes surgery and conventional chemotherapy. Lungs are most common site of metastasis followed by other bones [4]. Single drug regimen based chemotherapy is the mainstay treatment for OS. Despite great strides in OS treatment, no substantial improvement in OS therapy has taken place and remains elusive [5,6].

Cancer therapy based on single drug remains unsatisfying due to the complex microenvironment of cancer cells along with the drug resistance mechanisms [7]. In this regard, combination therapy has been considered as a promising strategy to improve the therapeutic efficiency and to minimize side effects [8]. The combination of two or more chemotherapeutic drugs may act synergistically towards the cancer cell suppression. The administration of anticancer drugs with two different molecular pathways can delay the cancer cell adaptation process and thereby reduce the cancer cell mutations. Chemotherapeutic drugs with same molecular pathway could function synergistically towards the better therapeutic efficacy and higher target selectivity [9,10]. In the present study, paclitaxel (PTX) and etoposide (ETP) were selected as a unique drug combination for the effective treatment of OS. PTX has been one of the widely used anticancer cytotoxic agents approved for the treatment of many malignancies. The cytotoxic action of PTX results from the interference in microtubule function. The destabilization of microtubules leads to depolymerization, M-phase cell cycle arrest, and cell death [11,12]. ETP on the other hand kills the cancer cells by stabilizing a covalent enzyme-DNA complex which is an important part of catalytic cycle of topoisomerase II. A permanent DNA strand breaks due to the accumulation of cleaved enzyme complexes, leading to mutagenesis, chromosomal translocation, and cell death [13,14]. As the individual efficacy of each of these two agents is very significant, we planned to evaluate the combination effect of PTX and ETP in this study.

Despite the significance of combination treatment, administration of cocktail combinational regimen did not result in better therapeutic efficacy [15]. The diverse pharmacokinetics, biodistribution, and membrane transport mechanism of two drugs would result in poor accumulation in cancer cells and attain suboptimal concentration at tumor site making dosing extremely difficult. Besides, it resulted in severe drug-related side effects in the clinical subjects [16]. Therefore, multiple chemotherapeutic drugs have to be entrapped in single nanocarriers such that it can release the active molecule in a predetermined manner. The ideal nanocarrier should possess high loading capacity for both the drug and should remain stable in blood circulation for prolonged period of time so that it can avail enhanced permeability and retention effect (EPR) [17]. A variety of nanoparticle-based delivery systems have been developed for the delivery of anticancer drugs. Among various nanocarriers, biodegradable polymer based poly(lactic-co-glycolic acid) (PLGA) nanoparticles was selected due to its excellent systemic characteristics and biodegradability. Several studies have reported that nanosized PLGA NP would be in the ideal range of EPR effect as well as to avoid reticuloendothelial system (RES) mediated clearance [18]. In the present study, PEGylated PLGA NP was developed for the delivery of multiple anticancer drugs.

The study was aimed at investigating the synergistic and enhanced apoptosis effect of unique combinational regimen. The most common treatment for osteosarcoma is the combination of therapeutic approaches and therefore in this study, two drugs were combined. We hypothesized that these two chemotherapeutic drugs when administered simultaneously would increase the therapeutic efficacy of cancer chemotherapy. For this purpose, PTX and ETP were co-encapsulated in PEGylated PLGA NP and evaluated its therapeutic efficacy. The dynamic light scattering analysis and morphology analysis were carried out to optimize the formulations. Drug release study was performed using dialysis method. We have evaluated the cytotoxic effect of individual and combined drugs in MG63 and Saos-2 osteosarcoma cell lines. The synergistic effect of combinational regimen was characterized by cell cycle analysis and apoptosis assay. This is the first study to depict the therapeutic efficacy of unique combination of PTX and ETP.

## Results and discussion

**Figure 1:**
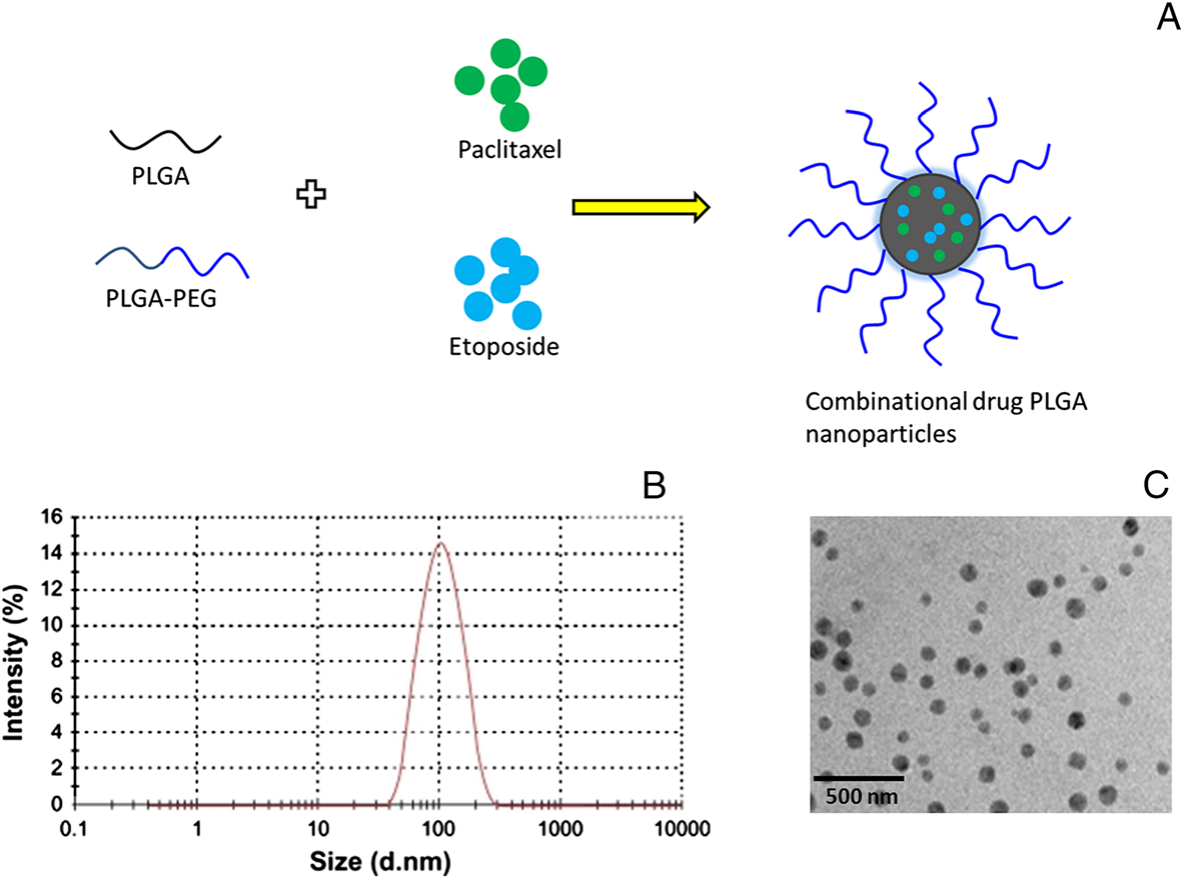
**Schematic illustration. (A)** Schematic representation of assembly of PLGA polymers and paclitaxel and etoposide towards the formation of drug-loaded nanoparticles. Schematic illustration of self-assembling process of polymeric nanoparticles. **(B)** Typical size distribution analysis of PLGA NP by dynamic light scattering technique **(C)** transmission electron microscope (TEM) imaging of drug-loaded PLGA NP.

Osteosarcoma (OS) is one of the most prevalent malignant bone cancers affecting adolescents aged between 10 and 24 years. In the clinical settings, implementation of chemotherapy greatly reduced the incidence of OS. However, chemotherapy suffers from the relapse and inefficient treatment, mainly due to the inability of single therapeutic modality to counter the tumor cells. Cancer therapy based on single drug remains unsatisfying due to the complex microenvironment of cancer cells along with the drug resistance mechanisms. . In this regard, combination therapy has been considered as a promising strategy to improve therapeutic efficiency and to minimize side effects. In the present study, paclitaxel (PTX) and etoposide (ETP) were selected as a unique drug combination for the effective treatment of OS. PTX has been known to interfere with microtubule function, while, ETP stabilizes the enzyme-DNA complex, resulting in mutagenesis, chromosomal translocation, and cell death. Nanoparticulate system has remarkably enhanced the therapeutic efficacy of anticancer drugs. The delivery system could result in more favourable pharmacokinetic profile, prolong blood circulation, and could potentially enhance the accumulation of drug in the cancer tissues via passive EPR effect. In this regard, PLGA nanoparticles have been reported to possess important characteristics such as excellent biocompatibility and biodegradability. Therefore, in the present study, PLGA NP has been used to incorporate two drugs. In order to prolong the systemic blood circulation time, PLGA NP was surface modified with PEG moiety that can endow the antifouling effect (Figure 1A).

### Physicochemical characterization of drug-loaded PLGA NP

The PLGA NP showed a high entrapment efficiency of 92.5 ± 5.6% for both the drug with a high loading capacity of 13.6 ± 2.8%. The high entrapment efficiency was attributed to the hydrophobic nature of anticancer drugs. Dynamic light scattering (DLS) technique was used to determine the particle size and size distribution. DLS showed that the average particle size of NP was around 100 ± 3.68 nm with uniform dispersion of particles (PDI, 0.14 ± 0.005) (Figure 1B). The nanosized particle along with PEG surface modification could potentially evade the macrophage-based clearance system and could preferentially accumulate in the cancer tissues via passive EPR effect (extravasate from the bloodstream) [19]. The PLGA NP showed a surface charge of +20 mV which is in the range of excellent colloidal stability. The size was further confirmed by TEM imaging (Figure 1C). The particle size from TEM (70 ± 4.5 nm) was slightly smaller than observed from DLS analysis (100 ± 3.68 nm). The discrepancies in sizes were due to that fact that TEM image was acquired on dry samples under vacuum whereas the DLS profile was obtained in an aqueous solution.

### In vitro release study

**Figure 2:**
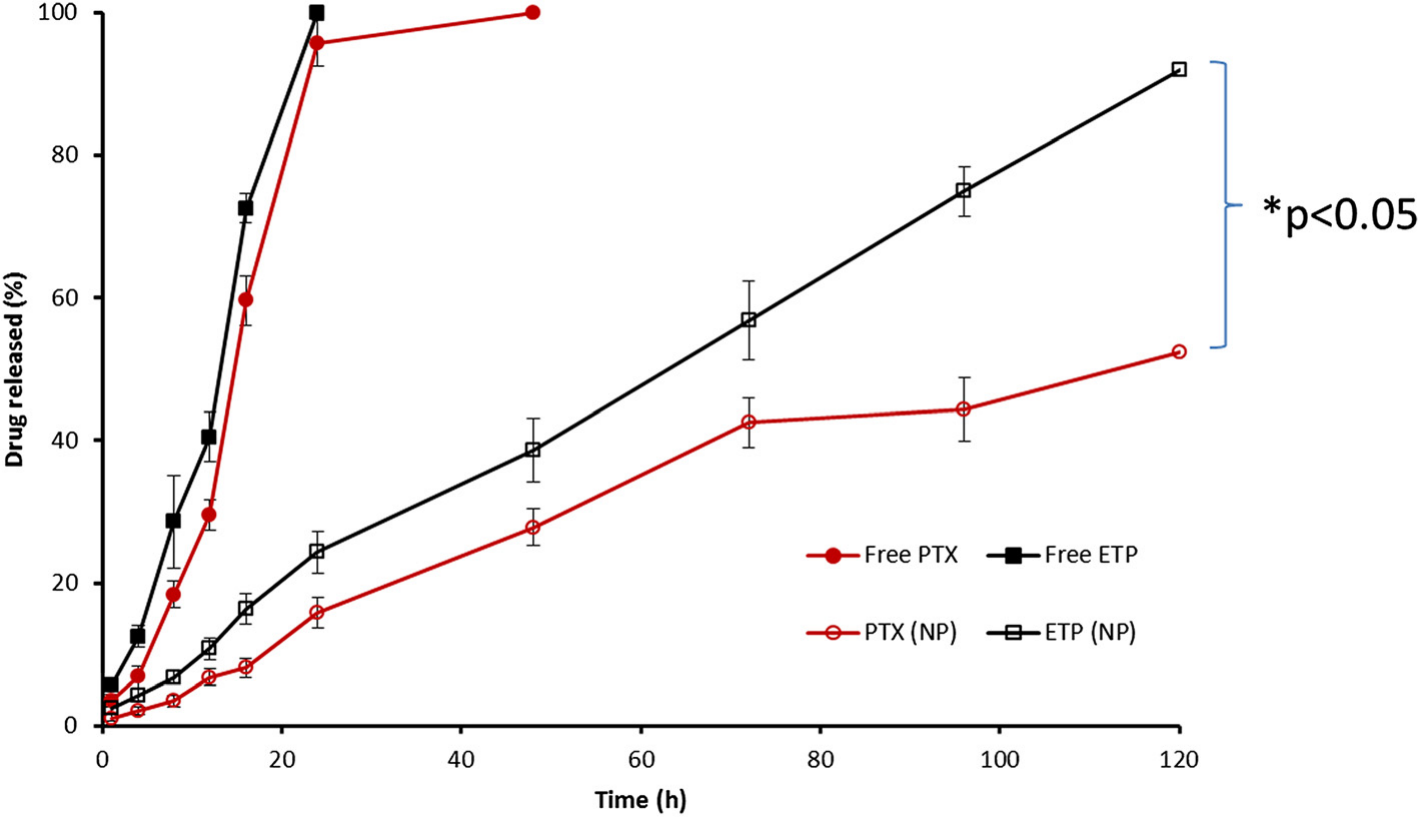
**Release profile of PTX and ETP from PTX-ETP/PLGA NP incubated at phosphate buffered saline (pH 7.4).** The samples were incubated at 37°C in a rotary shaker (100 rpm). The data are presented as mean ± SD (n = 3).

PTX (Log P ~ 3.96) and ETP (Log P ~ 1.16) were encapsulated into PLGA NP in 1:1 molar ratio. As shown in Figure 2, no initial burst release phenomenon was observed for both the drugs. The drugs (PTX and ETP) released in a sustained manner from the nanoparticulate systems up to 120 h study period. It has to be noted that, PTX and ETP has different release pattern with latter released relatively faster than that of PTX which was releasing slowly until the end of release study. For example, at the end of 24 h, approximately 15 ± 3.5% of PTX released comparing to that of 25 ± 2.6% ETP from PLGA NP. By 120 h, 90 ± 3.64% of ETP released from the NP, whereas only 50 ± 4.6% of PTX released during the same time period. The significant difference (p < 0.05) in release pattern among two drugs might be due to different hydrophobicity and position of drugs in the PLGA core. Nevertheless, a sustained release profile for both the drugs would be beneficial for the efficient cancer therapy. Furthermore, a sequential release of drugs would activate the cell apoptosis in cancer tissues.

### In vitro cytotoxicity analysis

**Figure 3:**
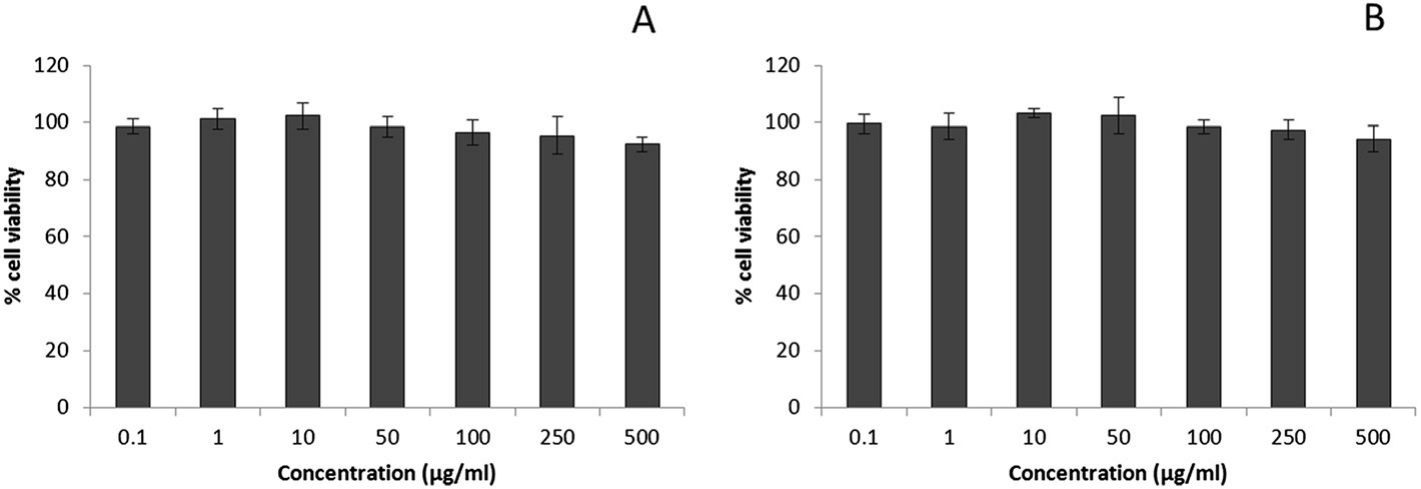
**In vitro cytotoxicity of blank polymeric nanoparticles at various concentrations against MG63 (A) Saos-2 cells (B).** The cells were incubated with blank PLGA NP for 24 h and the cytotoxicity assay was performed by MTT reagent.

The success of nanoparticulate drug delivery system relies on the safety and biocompatibility of delivery system in the in vivo environment. In the present study, biocompatibility of PEGylated PLGA nanoparticles were studied in MG63 and Saos-2 osteosarcoma cancer cells (Figure 3A, B). The results revealed that the nanoparticles were non-toxic and biocompatible at highest tested concentration. In both the cell lines, cell viabilities remained at more than 90% exhibiting its excellent safety profile. Such kind of non-toxic nature of polymers or nanoparticles is an ideal candidate for in vivo applications.

**Figure 4:**
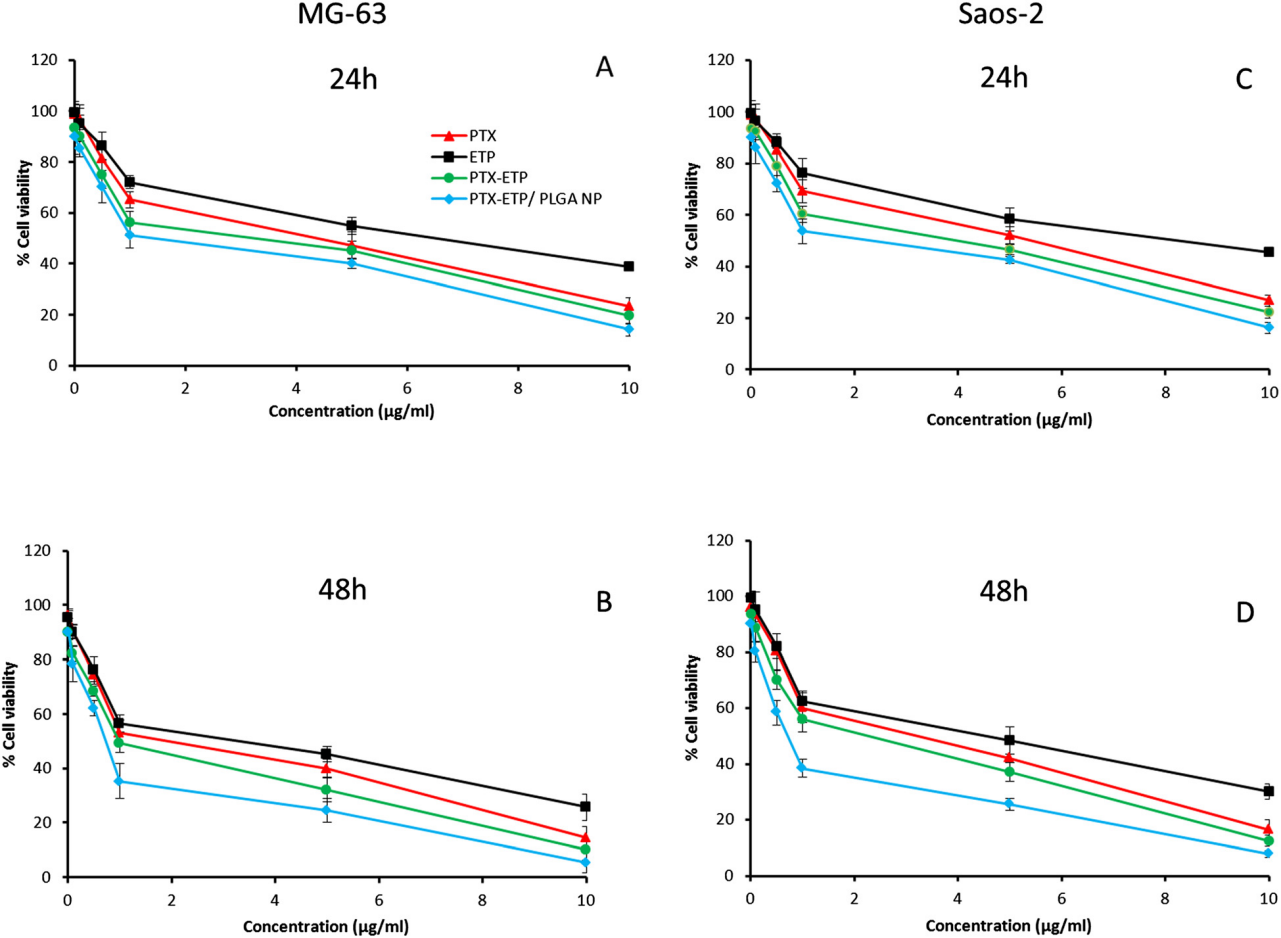
**In vitro cytotoxicity of free PTX, free ETP, free PTX/ETP, and PTX-ETP/PLGA NP against MG63 (A, B) Saos-2 cells (C, D) incubated for 24 and 48 h.** The cytotoxicity of formulations was evaluated by MTT assay. The data are presented as mean ± SD (n = 6).

Free PTX, free ETP, free PTX/ETP, and PTX-ETP/PLGA NP was tested in these cell lines. In MG63 cells, free drug and formulations exhibited a time-dependent and dose-dependent cytotoxicity (Figure 4A-D). Especially, PTX showed higher cytotoxic effect than ETP across all the concentration on different time points. As expected, combination of PTX/ETP substantially reduced the viability of cancer cells. Importantly, PTX-ETP/PLGA NP significantly arrested the growth of cancer cells. For example, PTX and ETP showed a cell viability of 65% and 72%, respectively, while combinational nanoparticle exhibited only 51% viability of cells indicating its superior anticancer effect [20]. The enhanced anticancer effect of PTX-ETP/PLGA NP was due to the controlled release of therapeutic drugs and high intracellular concentrations [21]. It is well known that free drug easily diffuse into the cell membrane whereas micellar drug takes a specific cellular internalization pathway and releases the drug in a systemized manner. Similar trend was observed in Saos-2 cancer cells however it was relatively less sensitive to anticancer drugs comparing to that of MG63 cells.

IC50 value was calculated to quantitate the amount of drug required to kill 50% of cancer cells. The IC50 value was calculated from GraphPad prism (version5) software. The IC50 values of free PTX, free ETP, free PTX/ETP, and PTX-ETP/PLGA NP were 4.56, 6.12, 3.82, and 1.45 μg/ml respectively in MG63 cells after 24 h incubation. In case of Saos-2 cells, IC50 values were 5.26, 7.15, 4.18, 1.98 μg/ml, respectively for all this formulations. The IC50 value clearly showed the superior performance of combinational nanoparticles. Moreover, it can be seen that Saos-2 cancer cell showed higher IC50 value than comparing to MG63 cancer cells. The combinational nanoparticulate formulations induced greater cancer cell killing potency than the cocktail free drugs. Moreover our results are consistent with the report that nanoparticles exhibit higher cytotoxicity owing to its higher cellular uptake [22].

### In vitro cellular uptake

**Figure 5:**
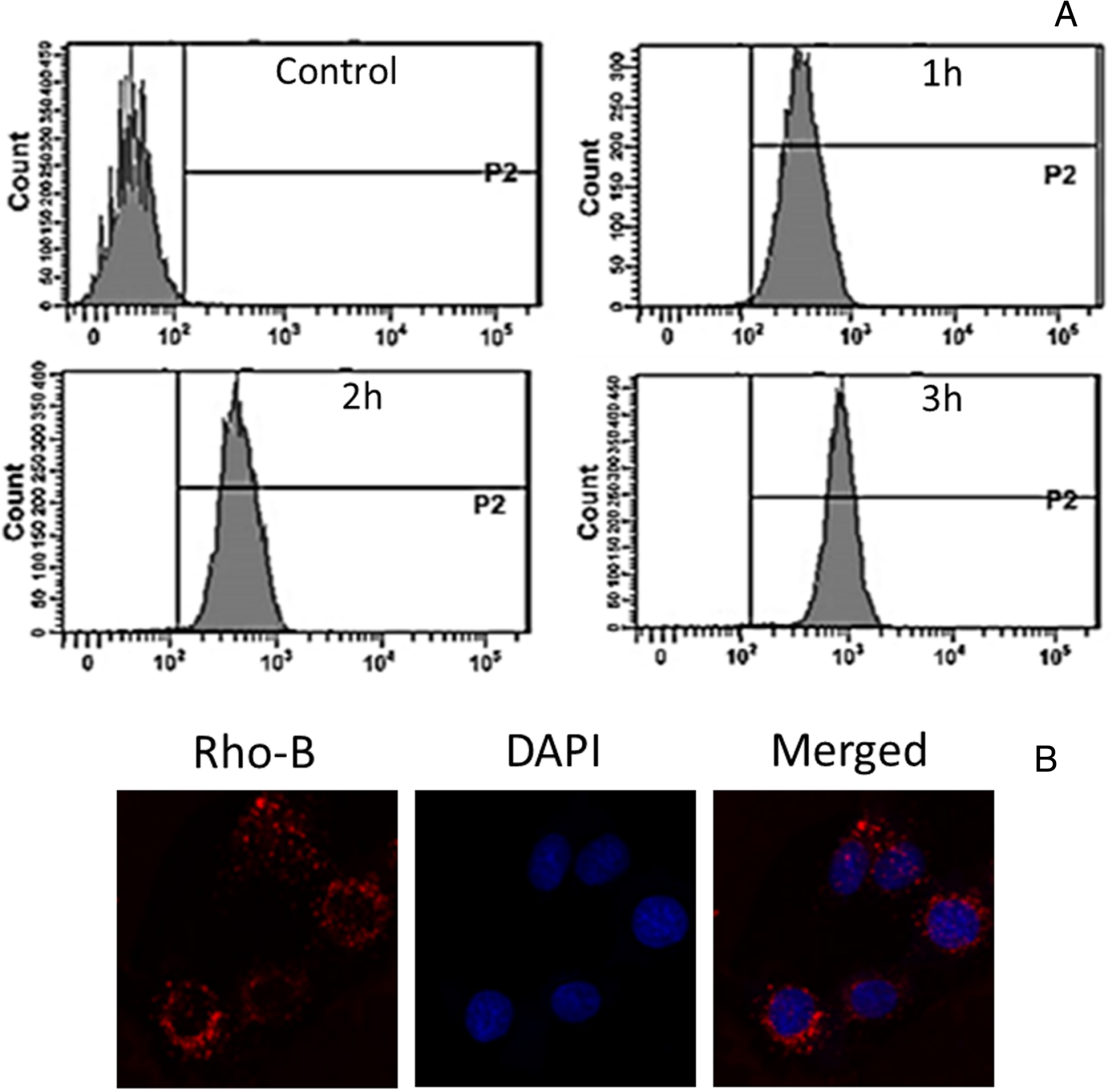
**Cellular uptake analysis. (A)** Flow cytometry analysis of in vitro cellular uptake of PLGA NP in a time dependent manner. The PLGA nanoparticle was loaded with Rhodamine B as a fluorescent dye. **(B)** Confocal microscopy images of MG63 cells. Cells were treated with PTX-ETP/PLGA NP and incubated for 1 h. Nuclei were stained with DAPI and Rho B fluorescence was observed in the cytoplasm.

The fluorescent labelled PLGA NP was incubated for 1 h, 2 h, and 3 h, respectively. As seen, NP showed a definite uptake within 1 h of incubation time. Consistently, cellular uptake increased with the increase in the incubation time of NP (Figure 5A). The results clearly demonstrate the time-dependent cellular internalization of NP which will increase the intracellular concentration of anticancer drugs leading to enhanced chemotherapeutic effect [23].

Cellular internalization and mechanism of cellular uptake was further investigated using confocal laser scanning microscopy. For this purpose, MG63 cells were incubated with Rho-B loaded PLGA NP at 37°C. As seen, nanoparticles were internalized via endocytosis process and accumulated in the cytoplasmic region (Figure 5B). In the merged image, presence of NP in the cytosol and DAPI stained nuclei were clearly differentiated. It could be expected that the NP after internalization will move from endosome towards lysosome or acidic compartment where it will release its therapeutic load [24]. After cellular internalization via endocytosis pathway, drug-loaded PLGA NP could greatly reduce the drug efflux from cytosol and it will enhance it interactions with the cellular components [25].

### Apoptosis assay

**Figure 6:**
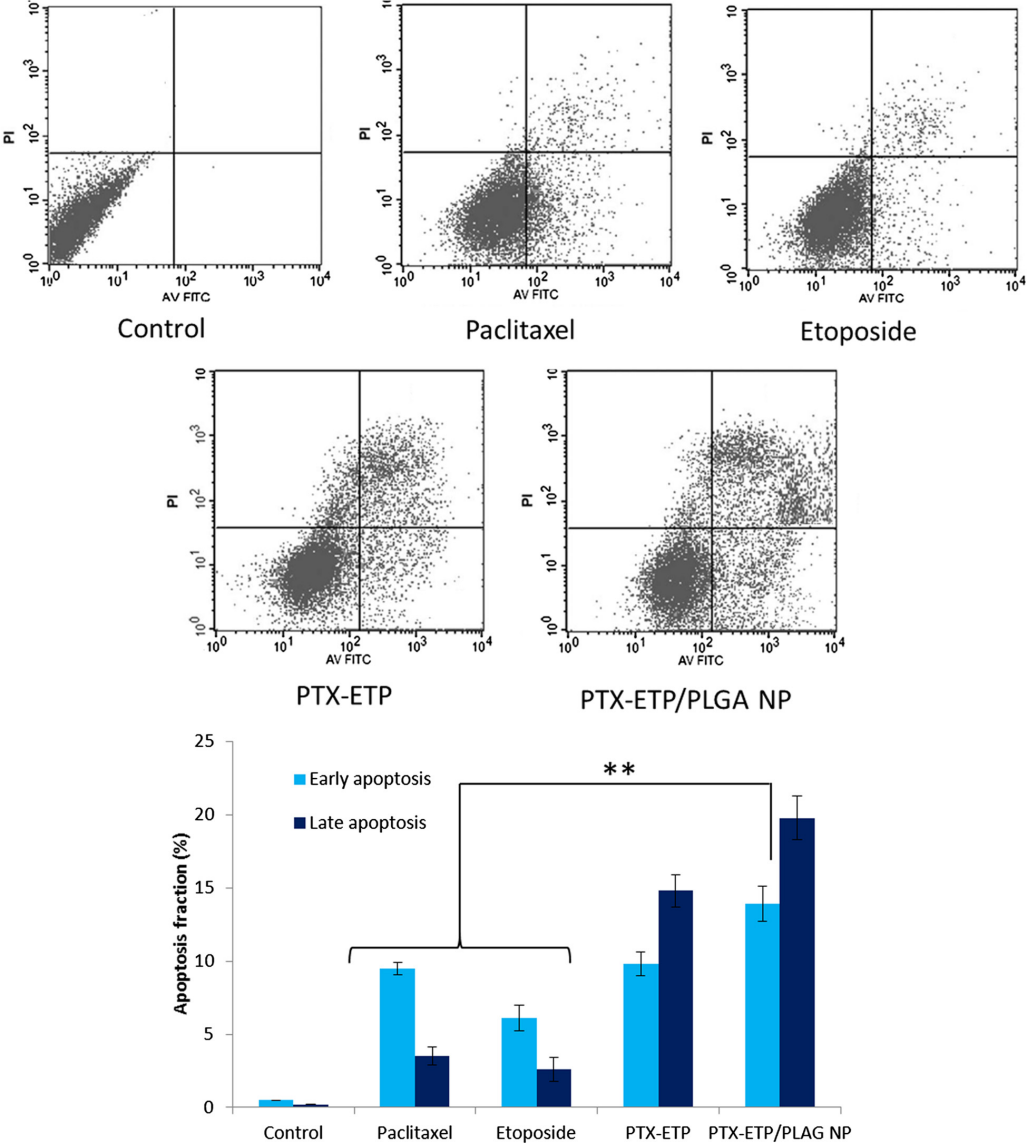
**Flow cytometer analysis of cell apoptosis using annexinV-FITC and PI staining.** The MG63 cells were exposed with free PTX, free ETP, free PTX/ETP, and PTX-ETP/PLGA NP at a concentration of 1000 ng/ml and incubated for 20 h. **p < 0.01 is the statistical difference between the combinational nanoparticles and individual free drugs.

Apoptosis is a programmed, physiological mode of cell death which plays an important role in tissue homeostasis. As a programmed cell death, cell apoptosis could be marked by a series of morphological and biochemical changes. The flipping of phosphatidylserine (PS) and loss in membrane potential are standard parameters for apoptosis detection. In this study, cell apoptosis was determined by Annexin V FITC and PI staining in MG63 cancer cells. The scatter plot has four quadrants: lower left quadrant (Q3) indicates viable cells (Annexin − ve, PI − ve), lower right quadrant (Q4) indicates early apoptotic cells (Annexin + ve, PI − ve), upper right quadrant (Q2) indicates late apoptotic cells (Annexin + ve, PI + ve) and upper left quadrant (Q1) indicates necrotic cells (PI + ve). In our experiment, we have treated MG63 cells with individual drugs as well as combinational drugs (with and without carrier). As seen (Figure 6), PTX induced apoptosis with much of cells in early apoptosis chamber and fewer cells in late apoptosis chamber. On the other hand, ETP has least effect on the cellular apoptosis with only few cells in apoptosis chamber. However when PTX and ETP were combined, it induced a significantly higher apoptosis than did the individual drugs. Approximately 15% of cells were in late apoptosis stage and 10% of cells in early apoptosis stage. As expected, PLGA NP remarkably induced a greater apoptosis of MG63 cells with more than 20% of cells in late apoptosis chamber (p < 0.01). The results clearly showed that combinational drugs improved the therapeutic index of each drug and resulted in superior anticancer effect [26]. Although, synergistic effect was seen in combined cocktail drugs, advantage of loading in nanoparticulate system would be to release the drug in an optimized sequential pattern [27].

### Cell cycle assay

**Figure 7:**
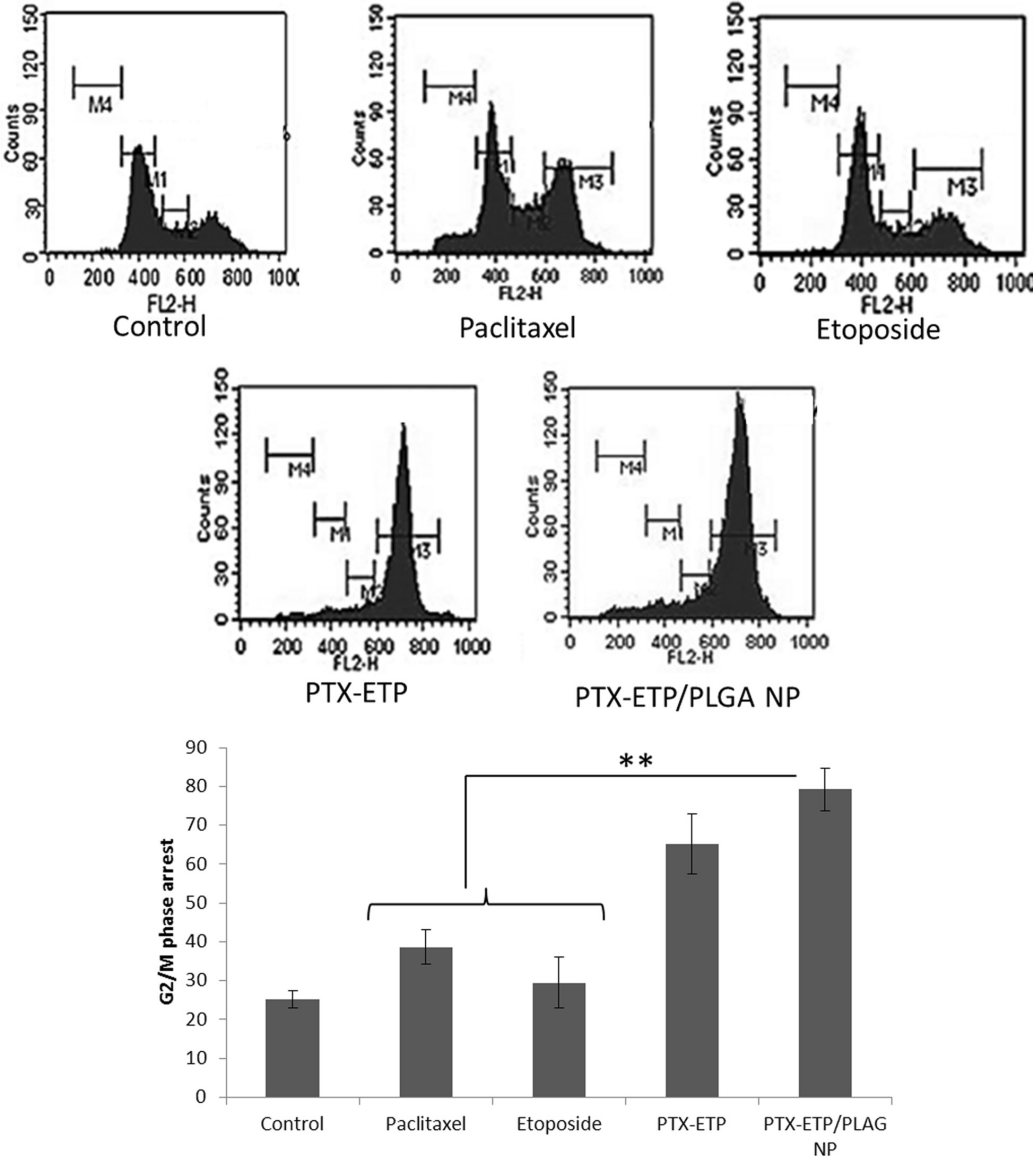
**Effect of free drug and drug loaded NPs on the cell cycle distribution of MG63 cells.** For cell cycle analysis, cells were treated with respective formulation and 24 h post treatment, stained with PI and RNAse before flow cytometer analysis. The region marked M1, M2, M3 and M4 represent G1, S, G2/M and G_0_ phase respectively, of the cell cycle. **p < 0.01 is the statistical difference between the combinational nanoparticles and individual free drugs.

Cell cycle progression is regarded as a hallmark of cancer progression and therefore effective strategy to inhibit cancer cell progression remains the primary strategy. In the present study, cell cycle progression has been analysed after treating with free PTX, free ETP, free PTX/ETP, and PTX-ETP/PLGA NP for 20 h (Figure 7). Results clearly showed that PTX arrests the cancer cell at G2/M phase of cell cycle, while ETP has relatively lesser effect on the cell cycle arrest. The combination of PTX/ETP however showed a significant (p < 0.01) cell cycle arrest G2/M phase when compared to native free drugs (individual). It has to be noted that nanoparticle encapsulation (PTX-ETP/PLGA NP) drugs arrested maximum number of cells in the same concentration as that of single drugs. The synergistic activity of combined drugs effectively checked the growth of cancer cells. Earlier, Park et al. reported that NP is more effective in controlling cell cycle progression than native free drugs [22]. Although the synergistic effect was observed with cocktail drugs however drug combination in nanoparticles would release the drug in a ratiometric manner to the tumor tissue, *in vivo*. The greater inhibitory effect of nanoparticle combination would be of great advantage during systemic cancer therapy [28].

## Conclusion

In conclusion, PTX and ETP co-loaded PLGA NP was successfully developed and characterized for its synergistic activity against osteosarcoma cancer cells. Various physicochemical and biological parameters were assessed to prove our concept. The particles were nanosized and exhibited a controlled sequential release pattern for both the drugs. The PLGA NP loaded drugs showed a potent anticancer effect in MG63 and Saos-2 cancer cells in a time and concentration dependent manner. Additionally, NPs showed an appreciable uptake in MG63 cells in a time-based manner. Co-delivery of anticancer drugs resulted in enhanced cell cycle arrest and apoptosis. The results clearly showed that combinational drugs remarkably improved the therapeutic index of chemotherapeutic drugs. The greater inhibitory effect of nanoparticle combination would be of great advantage during systemic cancer therapy. Taken together, multiple drug-loaded PLGA NP would be a promising cancer drug delivery system for the treatment of osteosarcoma.

## Materials and methods

### Methods

PLGA (50:50, MW 15000) was procured from the Shandong Institute of Medical Instruments (Shandong, China). PEG-PLGA was procured from Sigma-Aldrich, China. Paclitaxel was purchased from Xian Aladdin Biological Technology Co., Ltd, China. Etoposide was procured from Sigma-Aldrich, China. All other chemicals were reagent grade and used without further purifications.

### Preparation of drug-loaded PEGylated PLGA nanoparticles

PEGylated PLGA NP was prepared by solvent evaporation method. In brief, PTX and ETP (10% w/w of polymer) was dissolved in 1 ml of ethylacetate and 200 mg of PLGA and 20 mg of PEG-PLGA was separately dissolved in 1 ml of ethylacetate solution. Both the organic solutions were dissolved in 5% Pluronic 188 (pH 9.2) solution and sonicated for 2 min. 5 ml of 2% Pluronic 188 solution was added to the pre-formed primary w/o emulsions and sonicated for additional 2 min to form a w/o/w emulsions. The resulting double emulsion was in turn poured into 50 ml of 2% Pluronic 188 solution and mechanically stirred for 1 h at 1000 rpm. The drug loaded nanoparticles were washed, centrifuged, resuspended and stored at 4°C until further use.

### Particle size and zeta potential analysis

The particle size, size distribution, and zeta potential analysis were carried out by light scattering technique. Zeta Sizer 3000 HS (Malvern Instruments, UK) was used to measure the particle size at a fixed angle of 90° at 25°C. The formulations were suitably diluted with distilled water such that the mean count rate remains around 200. All measurements were performed in triplicate and presented as mean ± SD.

### Nanoparticles morphology

Transmission electron microscope (JEOL JEM-200CX) was used to characterize the morphology of nanoparticles. Briefly, liquid sample was placed in a carbon coated copper grid and counter stained with phosphotungistic acid, followed by air drying for 2 h. The samples were examined under acceleration voltage of 100 kV in conventional TEM mode.

### Drug loading

The drug loading capacity (DLC) and drug loading efficiency (DLE) was calculated using HPLC method. The drug formulations were filtered using Amicon centrifugal tube (MW cut off-3000). The filtrate was collected to determine the amount of the free drug (unentrapped) remains of the total amount of drug added. Few microliter of filtrate was injected into the HPLC column. Earlier, separate calibration curve has been built for PTX and ETP. The calibration equations were used to determine the amount of drug in the filtrate:

$$ \mathrm{D}\mathrm{L}\mathrm{C}\kern0.5em =\kern0.5em \left(\mathrm{Weight}\ \mathrm{of}\ \mathrm{drug}\ \mathrm{loaded}/\mathrm{weight}\ \mathrm{of}\ \mathrm{drug}-\mathrm{loaded}\ \mathrm{nanoparticles}\right) \times 100\% $$

$$ \mathrm{D}\mathrm{L}\mathrm{E}\kern0.5em =\kern0.5em \left(\mathrm{Weight}\ \mathrm{of}\ \mathrm{drug}\ \mathrm{loaded}/\mathrm{total}\ \mathrm{weight}\ \mathrm{of}\ \mathrm{drug}\ \mathrm{added}\right)\times 100\% $$

### In vitro drug release

The release study was performed by dialysis method. Briefly, PTX/ETP-loaded PLGA nanoparticles were freeze dried. The release study was performed in physiological media (pH 7.4; phosphate buffered saline) at 37°C. The freeze dried samples were dispersed in 1 ml of water and sealed in dialysis membrane tubing (MW cut off: 3000). The sealed membrane was placed in a 10 mL of release media and the whole assembly was in turn placed in shaking water bath maintained at 37°C. At selected time intervals, 1 ml of release media was withdrawn and replaced with equal volume of fresh medium. The amount of each drug released in the media was determined using HPLC method. Agilent 1100 equipped with a G1311A pump, a G1314A programmable diode array detector (DAD) and a G1313A auto-injector was used. C_18_ (250 mm × 4.6 mm ID, 5 μm) analytical column was used. Different mobile phase was used to characterize the concentration of individual drug released.

### Cell culture

MG63 and Saos-2 osteosarcoma cells were cultured in the DMEM growth medium supplemented with 10% foetal bovine serum, 1% -L-glutamine, and 1% penicillin/streptomycin mixture. The cells were grown in ambient conditions of 5% CO_2_ at 37°C. The cells were passaged when it reached 90% confluency.

### Cytotoxicity assay

The cytotoxicity assay was measured by 3-(4,5-dimethythiazol-2-yl)-2,5-diphenyl tetrazolium bromide (MTT) assay. It is based on the reduction of yellow MTT by mitochondrial succinate dehydrogenase. MTT enters the live cells and reduced into insoluble formazan complex. For this, MG63 and Saos-2 osteosarcoma cells were seeded at a density of 1 × 10^4^ in a 96-well plate. After 24 h, cells were exposed to blank polymer, free PTX, free ETP, PTX/ETP and PTX-ETP/PLGA NP at different dosing level. The cells were incubated for 24 and 48 h accordingly. At each time point, plate was removed and treated with 100 μl of MTT solution (5 mg/ml) to each 96-well plate and incubated for 4 h. The formed formazan crystals were extracted by adding DMSO and incubated for additional 30 min. The absorbance of each plate was read at 570 nm using a microplate reader (Thermo-Fisher, USA). All experiments were repeated 6 times.

### Cellular uptake by flow cytometry

BD FACS Calibur flow cytometer was used to determine the uptake potential of Rhodamine B Isothiocyanate (Rho-B) loaded PLGA NP in MG63 cancer cells. For this, 3 × 10^5^ cells were seeded on each well of 6-well plate and incubated overnight. The media was replaced with fresh media and treated with 50 μg/ml of PLGA NP (10 μg/ml equivalent of Rho-B) and cellular uptake was observed in a time-dependent manner (up to 3 h). Following incubation at 37°C, cells were washed twice with PBS, harvested, centrifuged, and resuspended in a growth medium. The process of washing and centrifugation was continued for 2 cycles. The cell suspension in 1 ml of PBS was used to determine the cellular internalization.

### Confocal laser scanning microscopy

Cellular uptake mechanism was investigated by means of confocal laser scanning microscopy. 3 × 10^5^ cells were seeded in a 6-well plate containing a cover slip. The cells were allowed to attach for 24 h. Following day, old media was replaced with new media and incubated with Rho-B-loaded PLGA NP for 2 h. The cells were washed twice with PBS and fixed with 4% paraformaldehyde for 20 min. The cells were washed again and the nuclei were stained with DAPI solution for 10 min. The cellular internalization process was evaluated using confocal microscopy.

### Cell cycle assays

The MG63 cells were seeded in a 12-well plate and the cells were allowed to attach for 24 h. Following day, old media was replaced with new media and exposed with free PTX, free ETP, free PTX/ETP, and PTX-ETP/PLGA NP for a period of 20 h. The cells were washed twice with PBS and harvested in the presence of trypsin. Cells were washed, centrifuged, and resuspended. Cells were suspended in 100 μl of PBS and 100 μl of reagent 1 (RNase (0.2 mg/mL) and Triton X-100) was added. After 5 min of vigorous stirring, 1 ml of reagent 2 (propidium iodide) was added, vortexed and incubated in dark for 60 min. The stained samples were then analysed using BD FACS Calibur flow cytometer.

### Determination of apoptosis assays

The apoptosis assay was carried out by annexin V/PI double stain assay. The MG63 cells were seeded in a 12-well plate and allowed to attach for 24 h to the walls. Following day, old media was replaced with new media and exposed with free PTX, free ETP, free PTX/ETP, and PTX-ETP/PLGA NP at a concentration of 1000 ng/ml and incubated for 20 h. The cells were washed twice with PBS and harvested in the presence of trypsin. The cells were centrifuged, and resuspended again in PBS. After final washing procedure, cells were redispersed in binding buffer and stained with FITC-annexin V and PI for 20 min. The cells were immediately analysed using BD FACS Calibur flow cytometer.

### Statistical analysis

Statistical analysis was performed by Student’s t-test for two groups, and one-way ANOVA for multiple groups. The significance level was set at a probability of P < 0.05. All results were reported as the mean ± standard deviation (SD).

## Abbreviations

PTX: Paclitaxel
ETP: Etoposide
NP: Nanoparticles
PEG: Polyethylene Glycol.

## References

[1] Longhi A, Errani C, De Paolis M, Mercuri M, Bacci G (2006). Primary bone osteosarcoma in the pediatric age: state of the art. Cancer Treat Rev.

[2] Dass CR, Ek ET, Contreras KG, Choong PF (2006). A novel orthotopic murine model provides insights into cellular and molecular characteristics contributing to human osteosarcoma. Clin Exp Metastasis.

[3] Ferrari S, Smeland S, Mercuri M, Bertoni F, Longhi A, Ruggieri P (2005). Neoadjuvant chemotherapy with high-dose Ifosfamide, high-dose methotrexate, cisplatin, and doxorubicin for patients with localized osteosarcoma of the extremity: a joint study by the Italian and Scandinavian Sarcoma Groups. J Clin Oncol.

[4] Geller DS, Gorlick R (2010). Osteosarcoma: a review of diagnosis, management, and treatment strategies. Clin Adv Hematol Oncol.

[5] Anninga JK, Gelderblom H, Fiocco M, Kroep JR, Taminiau AH, Hogendoorn PC (2011). Chemotherapeutic adjuvant treatment for osteosarcoma: where do we stand?. Eur J Cancer.

[6] Picci P, Mercuri M, Ferrari S, Alberghini M, Briccoli A, Ferrari C (2010). Survival in high-grade osteosarcoma: improvement over 21 years at a single institution. Ann Oncol.

[7] Woodcock J, Griffin JP, Behrman RE (2011). Development of novel combination therapies. N Engl J Med.

[8] Sun TM, Du JZ, Yao YD, Mao CQ, Dou S, Huang SY (2011). Simultaneous delivery of siRNA and paclitaxelviaa “Two in-One” micelleplex promotes synergistic tumor suppression. ACS Nano.

[9] Mauceri HJ, Hanna NN, Beckett MA, Gorski DH, Staba MJ, Stellato KA (1998). Combined effects of angiostatin and ionizing irradiation in antitumour therapy. Nature.

[10] Lane D (2006). Designer combination therapy for cancer. Nat Biotechnol.

[11] He L, Orr GA, Horwitz SB (2001). Novel molecules that interact with microtubules and have functional activity similar to Taxol. Drug Discov Today.

[12] Herbst RS, Khuri FR (2003). Mode of action of docetaxel—a basis for combination with novel anticancer agents. Cancer Treat Rev.

[13] Steward WP, Thatcher N, Edmundson JM, Shiu W, Wilkinson PM (1984). Etoposide infusions for treatment of metastatic lung cancer. Cancer Treat Rep.

[14] Baldwin EL, Osheroff N (2005). Etoposide, topoisomerase II and cancer. Curr Med Chem Anticancer Agents.

[15] Hu CM, Aryal S, Zhang L (2010). Nanoparticle-assisted combination therapies for effective cancer treatment. Ther Deliv.

[16] Lehar J, Krueger AS, Avery W, Heilbut AM, Johansen LM, Price ER (2009). Synergistic drug combinations tend to improve therapeutically relevant selectivity. Nat Biotechnol.

[17] Kolishetti N, Dhar S, Valencia PM, Lin LQ, Karnik R, Lippard SJ (2010). Engineering of self-assembled nanoparticle platform for precisely controlled combination drug therapy. Proc Natl Acad Sci U S A.

[18] Seju U, Kumar A, Sawant K (2011). Development and evaluation of olanzapineloaded PLGA nanoparticles for nose-to-brain delivery: in vitro and in vivo studies. Acta Biomater.

[19] Shimada T, Ueda M, Jinno H (2009). Development of targeted therapy with paclitaxel incorporated into EGF-conjugated nanoparticles. Anticancer Res.

[20] Perez EA, Buckwalter CA (1998). Sequence-dependent cytotoxicity of etoposide and paclitaxel in human breast and lung cancer cell lines. Cancer Chemother Pharmacol.

[21] Singh A, Talekar M, Tran TH, Samanta A, Sundaramb R, Amiji M (2014). Combinatorial approach in the design of multifunctional polymeric nano-delivery systems for cancer therapy. J Mater Chem B.

[22] Ramasamy T, Kim JH, Choi JY, Tran TH, Choi HG, Yong CS (2014). pH sensitive polyelectrolyte complex micelles for highly effective combination chemotherapy. J Mater Chem B.

[23] Blechinger J, Bauer AT, Torrano AA, Gorzelanny C, Bräuchle C, Schneider SW (2013). Uptake kinetics and nanotoxicity of silica nanoparticles are cell type dependent. Small.

[24] Pu Y, Chang S, Yuan H, Wang G, He B, Gu Z (2013). The anti-tumor efficiency of poly(L-glutamic acid) dendrimers with polyhedral oligomeric silsesquioxane cores. Biomaterials.

[25] Wei R, Cheng L, Zheng M, Cheng R, Meng F, Deng C (2012). Reduction responsive disassemblable core-cross-linked micelles based on poly (ethylene glycol)-b-poly (n-2-hydroxypropyl methacrylamide)-lipoic acid conjugates for triggered intracellular anticancer drug release. Biomacromolecules.

[26] Anitha A, Deepa N, Chennazhi KP, Lakshmanan VK, Jayakumar R (1840). Combinatorial anticancer effects of curcumin and 5-fluorouracil loaded thiolated chitosan nanoparticles towards colon cancer treatment. Biochim Biophys Acta.

[27] Park JS, Han TH, Lee KY, Han SS, Hwang JJ, Moon DH (2006). N-acetyl histidineconjugated glycol chitosan self-assembled nanoparticles for intracytoplasmic delivery of drugs: endocytosis, exocytosis and drug release. J Control Release.

[28] Ramasamy T, Ruttala HB, Choi JY, Tran TH, Ku SK, Choi HG (2015). Engineering of a lipid-polymer nanoarchitectural platform for highly effective combination therapy of doxorubicin and irinotecan. Chemical Communications..

